# Acute kidney rejection after anti-SARS-CoV-2 virus-vectored vaccine—case report

**DOI:** 10.1038/s41541-022-00445-5

**Published:** 2022-03-02

**Authors:** Matej Vnučák, Karol Graňák, Monika Beliančinová, Miloš Jeseňák, Katarína Kajová Macháleková, Jakub Benko, Matej Samoš, Ivana Dedinská

**Affiliations:** 1 Transplantation Centre, University Hospital Martin and Jessenius Medical Faculty of Comenius University, Martin, Slovakia; 2 Department of Children and Adolescents, University Hospital Martin and Jessenius Medical Faculty of Comenius University, Martin, Slovakia; 3 Department of Pathology, St. Elizabeth Cancer Institute Hospital, Bratislava, Slovakia; 4 1st Department of Internal Diseases, University Hospital Martin and Jessenius Medical Faculty of Comenius University, Martin, Slovakia

**Keywords:** Acute kidney injury, Risk factors

## Abstract

COVID-19 infection remains a threat to the health systems of many countries. Potential success in the fight against the COVID-19 pandemic is the vaccination of high-risk groups, including patients with end-stage kidney disease (ESKD) and after solid organ transplantation (SOT). Immunosuppression in kidney transplant recipients can also reduce the immunogenicity of SARS-CoV-2 vaccines (varied by vaccine platform), available data suggest that they are efficacious in approximately 50–70%, compared to non-transplant situations. In this paper, we present a newly developed acute humoral and cellular rejection with acute allograft failure and need of hemodialysis 14 days after administration of the adenovirus vectored SARS-CoV-2 vaccine (AstraZeneca; CHADOx1, AZD1222). This occurred in a patient who previously had an asymptomatic COVID-19 infection. Case reports of acute allograft rejection after vaccination against SARS-CoV-2 can help stratify risk groups of patients who develop hyperimmune reactions. However, it is also possible that those with a previous mild primary COVID-19 infection may also develop acute allograft rejections upon COVID-19 re-infection.

## Introduction

The Coronavirus pandemic of 2019 (COVID-19) is a serious medical problem with the burden on advanced health systems increasing the cost of health care provided. Vaccination has emerged as a key tool for controlling the ongoing pandemic. Individuals who have undergone kidney transplants have been identified as high-risk populations and prioritized for vaccination, but have been excluded from major severe acute respiratory syndrome coronavirus 2 (SARS-CoV-2) vaccine clinical trials. COVID-19 has had an enormous effect on kidney and another solid organ transplant (SOT) recipients^1^. The case fatality rate of COVID-19 among patients with end-stage kidney disease (ESKD) is between 20 and 30% and the adjusted relative rate of death was 30% higher among kidney transplant recipients (KTR) and 17% higher among patients undergoing dialysis^2^. Because of increased COVID-19 associated morbidity and mortality in KTR, it has been proposed that the benefit of selected SARS-CoV-2 vaccines outweighs the risk of vaccination^3^. There has been a rapid vaccine development in response to the pandemic, in particular with the mRNA vaccines (Pfizer-BioNTech, Moderna) and viral vector-based vaccines (Sputnik V, AstraZeneca/Oxford, Janssen/Ad26.COV.2). Despite the theoretical concerns with replication-deficient viral vector-based vaccines, immunosuppression is not considered as a contraindication to their use^4^. Immunosuppression in KTR can also reduce the immunogenicity of SARS-CoV-2 vaccines with relative seroconversion rates of approximately 50–70% compared to non-transplant situations^5^.

## Case report

A 25-year-old woman with a kidney transplant due to diabetic kidney disease was referred to the Transplantation Center of the University Hospital Martin (TC UHM) due to a decline in her renal function. Her history includes end-stage kidney disease from diabetes mellitus type 1, hypertension, and autoimmune thyroiditis.

In November 2016 she underwent successful primary deceased donor kidney transplantation (KT). It was a blood-group compatible male donor with a three-year age difference and there were 1-1-2 mismatches in the human leukocyte antigen (HLA)-A, B, and DR group. HLA of the recipient was: A3, A68, B13, B35, DR4, DR8, DR53, Cw4, Cw6, DQ4, and DQ8. A cross-match assay by using a fluorescence-activated cell sorter was performed prior to transplantation and was negative in both the T and B components. As per our induction protocol, she received anti-thymocyte globulin (ATG) with a cumulative dose of 3.5 mg/kg of body weight as well as 500 mg of methylprednisolone prior to and the day after her KT. The maintenance immunosuppression administered consisted of tacrolimus at 0.2 mg per kg of body weight, mycophenolate sodium 720 mg twice a day, and prednisone 20 mg daily. The surgery was performed without complications; she had primary onset of graft function, adequate daily diuresis, and a decrease in her serum creatinine. Her graft function was stable, and she did not report any difficulties. She had no de novo done specific antibodies (dnDSA) when measured the first and second year after KT.

**Fig. 1:**
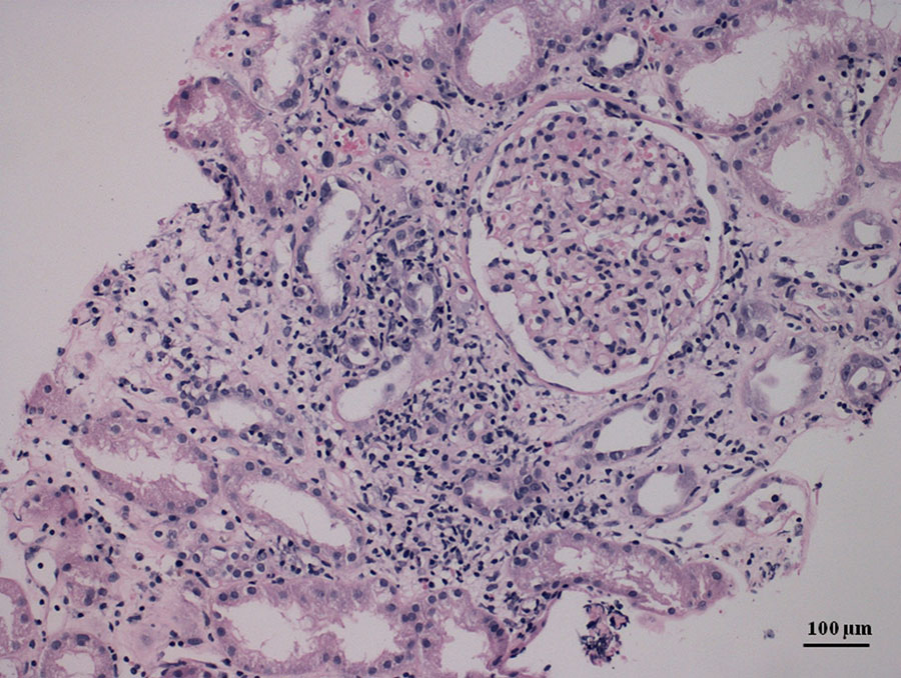
Glomerulitis and stromal infiltrate edema. Detail of a rejection infiltrates with an abundant admixture of plasma cells (hematoxylin–eosin, magnification: 400×, scale bar: 50 µm).

**Fig. 2:**
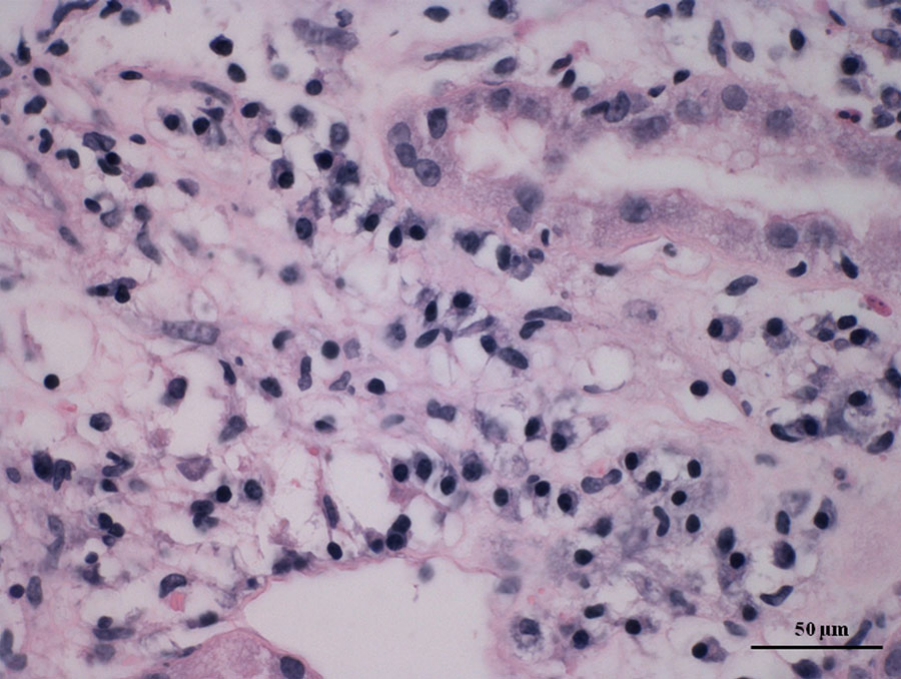
Abundant plasma cells in infiltrate. Clear image with glomerulitis, rejection infiltrate, and stromal edema (hematoxylin–eosin, magnification: 100×, scale bar: 100 µm).

In December 2019 she was diagnosed with asymptomatic COVID-19 infection. She had random reverse transcription-polymerase chain reaction (RT-PCR) testing and she did not require hospitalization, discontinuation, or reduction of immunosuppression, with her serum level of creatinine at 84 µmol/l (normal range 53–97 µmol/l), and proteinuria at 0.180 g/day (normal range: less than 150 mg/day). Four months later, on 11 April 2021, she was vaccinated with the COVID-19 adenovirus-vectored vaccine, AstraZeneca (ChAdOx1, AZD1222). Fourteen days later she was admitted to the intensive care unit of the Internal Department at the regional hospital. She complained of fatigue, general weakness, and vomiting, with the inability to eat or drink. She denied abdominal pain and diarrhea. She did not have fever, cough, or dyspnea. At the initial examination, her blood pressure was 135/75 mmHg, heart rate 96 beats per minute, and temperature 36.6 degrees centigrade. Her physical examination was normal including clear lungs to auscultation, soft abdominal exam with normoactive bowel sounds, and grossly normal neurology exam without asterixis. Laboratory values upon admission to the hospital are shown in Table 1. Chest radiograph did not show any abnormalities or signs of pneumonia. An abnormal gas pattern was present in the abdominal image, but without signs of ileus. The abdominal ultrasound did not show any abnormalities on hepato-pancreato-biliary system. The transplanted kidney was of normal shape and size with normal width of parenchyma, with preserved echogenicity and blood flow, normal resistance index with low resistance curves. Hydronephrosis of the transplanted kidneys was not detected. Esophagogastroduodenoscopy was negative; there were no signs of inflammation, ulcers, or Helicobacter pylori infection. Qualitative multiplex RT-PCR for diagnosing gastrointestinal viruses (adenovirus, astrovirus, norovirus, and rotavirus) was performed and was negative. Cytomegalovirus, adenovirus infection was ruled out by negative PCR of the blood. PCR of Ebstein-Barr virus and BK virus from blood were also negative. PCR test for COVID-19 infection was also negative. The patient was rehydrated, her metabolic acidosis was corrected, and her immunosuppression was continued. Her serum creatinine levels improved but remained abnormal; she was then referred to the Transplant Center of Nephrology in Martin. Anti-SARS-CoV-2- Spike protein antibodies were positive (>250 U/ml), as well as antibodies against N-protein of SARS-CoV-2 (27.440 U/ml). A percutaneous needle graft biopsy was performed. Histological examination revealed acute cellular rejection IB with moderate to severe tubulitis and focal tubular destruction, interstitially with dispersed round cells (40–50%) with an admixture of abundant plasma cells. It also revealed a mild degree of interstitial fibrosis and tubular atrophy (IFTA I). acute humoral rejection with C4d positivity (Grade II—ATN-like with signs of scattered thrombosis) (Figs. 1 and 2). Using the Luminex method we verified the presence of dnDSA: anti-HLA B57 (1700 mean fluorescence intensity [MFI]) and anti-HLA DQ2 (6460 MFI).

**Table 1:** Laboratory values upon admission to the hospital.

	Values	Normal range
Glucose	4.8–13.7	4.0–5.6 mmol/l
Creatinine	952	53–97 µmol/l
Urea	26.7	2.0–6.7 mmol/l
Sodium	136	135–145 mmol/l
Potassium	4.9	3.5–5.1 mmol/l
Chloride	106	98–106 mmol/l
Total protein	64.6	66–83 g/l
Albumin	30.9	35–50 g/l
Amylase	0.36	0.13–0.88 μkat/l
Lipase	0.3	0.22–1.00 μkat/l
AST	0.21	0.17–0.60 μkat/l
ALT	0.23	0.20–0.60 μkat/l
ALP	1.0	0.58–1.74 μkat/l
GMT	0.23	0.15–0.65 μkat/l
Total bilirubin	12.3	<21.0 µmol/l
Direct bilirubin	4.1	<5.1 µmol/l
pH	7.244	7.35–7.45
Base deficit	14.8	(−3)–(+3)
HCO3^-^	13.2	22–26 mmol/l
pO_2_	9.19	11.1–14.4 kPa
pCO_2_	3.35	4.3–6.0 kPa
WBC	13.7	4–10 × 10^9^/l
Hemoglobin	105	120–150 g/l
C-reactive protein	25.1	<5.0 mg/l
Procalcitonin	0.365	0.02–0.10 µg/l
Urine sediment	Bacteria, nitrite: 0 WBC: 3 RBC: 1 Epithelial cells: 0	Bacteria, nitrite: 0 WBC < 5 RBC < 5 Epithelial cells < 5
Urine culture	Negative	

*AST* aspartate aminotransferase, *ALT* alanine transaminase, *ALP* alkaline phosphatase, *GMT* gamma-glutamyl transferase, *RBC* red blood cells, *WBC* white blood cells

**Fig. 3:**
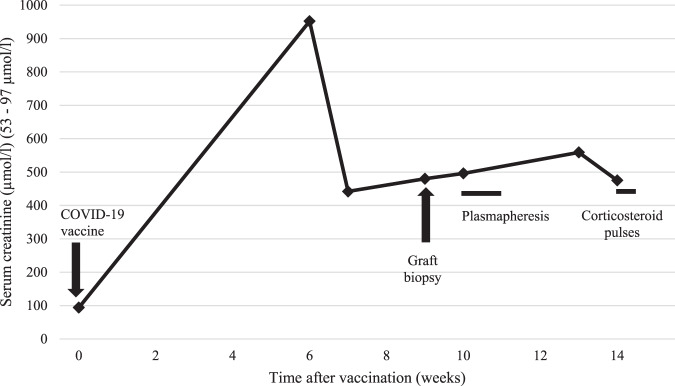
Serum levels of creatinine. It reflects the course of creatinine after COVID-19 vaccination (time 0), in time of graft biopsy and after the treatment (plasmapheresis, corticosteroid pulses).

We initiated combined antirejection therapy consisting of intravenous immunoglobulins with a cumulative dose 55 g (0.5 g/kg first day and 0.2 g/kg third and fifth day), 3 sessions of plasmapheresis, and administration of corticosteroids with a cumulative dose of 3 g. Additionally, she received a loop diuretic to enhance daily diuresis, which was maintained at a minimum of 2 l/day. We observed a mild decrease of serum creatinine, but increased serum levels of urea with the need for hemodialysis. The course of creatinine serum levels is shown in Fig. 3. Her dnDSA levels in class 1 were negative and class 2 dropped to 804 MFI. Her urine culture grew in *Escherichia coli*. Treatment with anti-CD20 monoclonal antibody (Rituximab) was held due to the increased inflammatory markers (CRP, leukocytosis) and high risk of septic complications.

Subject in submitting case report has given written informed consent for publishing.

## Discussion

The COVID-19 pandemic remains a global threat to the general population. KTR and patients with ESKD are at a high risk of morbidity and mortality as immunosuppression may increase the viral load of SARS-CoV-2 and prolong the duration of viral shedding and transmissibility. On the other hand, calcineurin inhibitors (CNI) play an important role in blocking key signal pathways of the T-cell antigen receptor with inhibition of interleukin-2 production. Therefore, CNI can inhibit secondary hemophagocytic lymphohistiocytosis as the cause of the cytokine storm in SARS-CoV-2^5^. Immunosuppression-related factors were not associated with increased mortality in SOT recipients, while comorbidities such as chronic heart failure, obesity, age older than 65 years, chronic lung disease were associated with increased mortality. Due to these factors, this class of patients should be prioritized for vaccination^1^. Immunosuppression in KTR can also reduce the immunogenicity of SARS-CoV-2 vaccines (varied by vaccine platform); KTR has relative seroconversion rates of approximately 50–70% compared to nontransplant patients^6^. In the post-transplant period, patients older than 65 years, more recent KT, use of mycophenolate, and mammalian target of rapamycin inhibitors are associated with decreased serologic response to influenza vaccines^7^. Vaccine antigen or adjuvants can induce a generalized systemic inflammation response or could promote allograft-directed immune responses^8^. Adenovirus vectors can trigger a potent immune response through complement activation and induce a diverse cytokine response^9^. Three cases of transverse myelitis were reported after ChAdOx1 NCoV-19 (AZD1222) booster vaccination. They were described as potentially related to the vaccination, later they were considered as idiopathic spinal cord demyelination or pre-existing multiple sclerosis. The relationship between the vaccine and acute transverse myelitis remained possible in only one of the cases^10^. It is known that natural infection can result in autoimmune disorders; for example, the relative risk of Guillain-Barre syndrome development is tenfold higher after natural flu than vaccination^11^. Most cases of acute kidney injury (AKI) associated with COVID-19 infection are the result of hemodynamic instability, cytokine related-injury, and the dysfunction of the coagulation cascade. The risk of graft rejection may result from the widespread practice of reducing anti-rejection therapy. However, performing graft biopsy and diagnosing acute cellular or humoral rejection is difficult in critically ill kidney transplant recipients with COVID-19 infection. In the study of Bajpai et al., 11% of KTR with COVID-19 infection had AKI with only 40% complete recovery of graft function. In this report, only 11 kidney biopsies were performed: 80% revealed acute tubular necrosis with two patients of 11 were diagnosed with acute cellular rejection and only one patient developed an elevated dnDSA level. Anti-metabolite was reduced in 84.6% of patients and CNI therapy was withdrawn or reduced in 62.5% of patients^12^.

It is important to highlight that the graft function in our case report remains unchanged after asymptomatic COVID-19 infection. Generally, vaccine adjuvants are used to enhance vaccine immunogenicity and have the potential to induce acute allograft rejection^9^. Concerns arose from the observation of high incidence of anti-HLA (only a small fraction was donor-specific antibodies) antibodies in KTR who received influenza A(H1Na1) pdm09 vaccine in 2009, which contained the squalene-based AS03 adjuvant system^6,7^. Several recombinant spike protein SARS-CoV-2 vaccines contain adjuvant (AS03, the novel Matrix M1 adjuvant), such as Novavax/NVX-CoV2373. However, the viral-vectored and mRNA vaccines do not generally contain adjuvants. One case report of acute cellular kidney rejection was reported after the second dose of mRNA vaccine (BNT162b2, Pfizer-BioNTech) to date^13^.

Some data show the low effectiveness of viral vectors vaccines due to prior existing immune response to the vector^14^. Our patient overcame asymptomatic COVID-19 infection 5 months prior to vaccination as verified by positive antibodies against the N-protein of SARS-CoV-2. The study of Wang et al. suggests that robust enhancement of B cell memory and serologic responses achieved with mRNA vaccination in patients after COVID-19 infection will provide high levels of protection against variants without the need to modify existing vaccines^15^. Therefore, vaccination was strongly recommended. The acute humoral and cellular rejection presented in the case report can be explained as a hyperimmune response to the first dose of a viral vector vaccine. This is represented by high levels of antibodies against the spike protein SARS-CoV-2 in a patient with autoimmune disease and previous asymptomatic COVID-19 infection. On the other hand, the risk of a hyperimmune reaction after COVID-19 re-infection is highly possible as well.

Despite the development of many types of vaccines, COVID-19 infection remains a threat to the health systems of many countries. Potential success in the fight against the COVID-19 pandemic includes the vaccination of high-risk groups such as patients with ESKD and after solid organ transplantation. In patients after solid organ transplantation, there is a clear benefit of vaccination against COVID-19, even with a potential lower immune response. Case reports of acute allograft rejection after vaccination against SARS-CoV-2 can help us stratify the risk group of patients with hyperimmune reactions after SARS-CoV-2 vaccines (mainly young patients with autoimmune diseases) but it is possible that such a group of patients may develop acute allograft rejection upon COVID-19 re-infection, especially in those with a mild form of primary COVID-19 infection. Therefore, it is worth considering monitoring graft function after vaccination against COVID-19 by examination of serum creatinine, proteinuria, and dnDSA.

### Reporting summary

Further information on research design is available in the Nature Research Reporting Summary linked to this article.

## Supplementary information

REPORTING SUMMARY

## Data Availability

No datasets were generated or analyzed during the current study.

## References

[1] Kates OS (2021). UW COVID-19 SOT Study Team: COVID-19 in solid organ transplant: a multi-center cohort study. Clin. Infect. Dis.

[2] Weinhandl ED (2021). Initial effects of COVID-19 on patients with ESKD. JASN.

[3] National Kidney Registry: COVID letter. https://www.kidneyregistry.org/pages/p680/COVID (2020).

[4] Aslam S (2021). COVID-19 vaccination in our transplant recipients: the time is now. J. Heart Lung Transplant..

[5] Mehta P (2020). HLH across speciality collaboration, UK. COVID-19: consider cytokine storm syndromes and immunosuppression. Lancet.

[6] Danziger-Isakov L, Kumar D (2019). AST ID Community of Practice. Vaccination of solid organ transplant candidates and recipients: guidelines from the American society of transplantation infectious diseases community of practice. Clin. Transplant..

[7] Haddadin Z (2021). Alternative strategies of posttransplant influenza vaccination in adult solid organ transplant recipients. Am. J. Transpl..

[8] Heldman MR, Limaye AP (2021). SARS-CoV-2 vaccines in kidney transplant recipients: will they be safe and effective and how will we know?. JASN.

[9] Ahi YS, Bangari DS, Mittal SK (2011). Adenoviral vector immunity: its implications and circumvention strategies. Curr. Gene Ther..

[10] De Haan P, Van Diemen FR, Toscano MG (2021). Viral gene delivery vectors: the next generation medicines for immune-related diseases. Hum. Vaccine Immunother..

[11] Münz C, Lünemann JD, Getts MT, Miller SD (2009). Antiviral immune responses: triggers of or triggered by autoimmunity?. Nat. Rev. Immunol..

[12] Bajpai D (2021). Recovery of kidney function after AKI because of COVID-19 in kidney transplant recipients. Transpl. Int..

[13] Del Bello A (2021). Acute rejection after anti-SARS-CoV-2 mRNA vaccination in a patient who underwent a kidney transplant. Kidney Int..

[14] Custers J (2021). Vaccines based on replication incompetent Ad26 viral vectors: standardized template with key considerations for a risk/benefit assessment. Vaccine.

[15] Wang Z (2021). Naturally enhanced neutralizing breadth against SARS-CoV-2 one year after infection. Nature.

